# Application of Genetic Algorithms for Pixel Selection in MIA-QSAR Studies on Anti-HIV HEPT Analogues for New Design Derivatives

**DOI:** 10.22037/ijpr.2019.1100731

**Published:** 2019

**Authors:** Zohreh Doroudi, Ali Niazi

**Affiliations:** a *Department of Chemistry, Arak Branch, Islamic Azad University, Arak, Iran. *; b *Department of Chemistry, Central Tehran Branch, Islamic Azad University, Tehran, Iran.*

**Keywords:** Multivariate image analysis, Genetic algorithms, Partial least square, Principal Component Regression, Variable selection, 1-[2-hydroxyethoxy)methyl]-6-(phenylthio)thymine

## Abstract

Quantitative structure-activity relationship (QSAR) analysis has been carried out with a series of 107 anti-HIV HEPT compounds with antiviral activity, which was performed by chemometrics methods. Bi-dimensional images were used to calculate some pixels and multivariate image analysis was applied to QSAR modelling of the anti-HIV potential of HEPT analogues by means of multivariate calibration, such as principal component regression (PCR) and partial least squares (PLS). In this paper, we investigated the effect of pixel selection by application of genetic algorithms (GAs) for the PLS model. GAs is very useful in the variable selection in modelling and calibration because of the strong effect of the relationship between presence/absence of variables in a calibration model and the prediction ability of the model itself. The subset of pixels, which resulted in the low prediction error, was selected by genetic algorithms. The resulted GA-PLS model had a high statistical quality (RMSEP = 0.0423 and R^2 ^= 0.9412) in comparison with PCR (RMSEP = 0.4559, R^2 ^= 0.7929) and PLS (RMSEP = 0.3275 and R^2 ^= 0.0.8427) for predicting the activity of the compounds. Because of high correlation between values of predicted and experimental activities, MIA-QSAR proved to be a highly predictive approach.

## Introduction

Acquired immunodeficiency syndrome (AIDS) is a disease of the human immune system caused by infection with human immunodeficiency virus (HIV). AIDS was first recognized in 1981 and its cause—HIV infection—was identified in the early part of the decade. The World Health Organization (WHO), in its reports, has said that AIDS has killed more than 25 million people since 1981, which is the most destructive among all pandemics in history. There were approximately 36.7 million people living with HIV at the end of 2015.

These alarming numbers have actuated the scientific community to search for therapies in the treatment of HIV-positive patients, and the development of novel and potent inhibitors for the treatment of HIV-1 infection has become the primary focus in this field. Researchers have investigated various ligands. Biologists, chemists, and researchers, in general, are continuously looking for new entities having high potency against the HIV virus. Such ligands may be properly developed using computer-assisted methods, known as *in-silico* QSAR (quantitative structure-activity relationship) procedures, which may be classified as ligand- and receptor-based approaches (1): 1-[2-Hydroxyethoxy) methyl]-6-(phenylthio)-thymines (HEPT), as shown on Table 1. HEPT forms the non-nucleoside RT inhibitors (NNRTI) series do not target an active site of polymerase, but rather the enzyme allosteric site. The interactions of these compounds with reverse transcriptase (RT) have been thoroughly investigated and the crystal structures of several ligand-enzyme complexes have been determined (2, 3).

Today, researchers use computational techniques to estimate the activity of designed molecules in order to accelerate the synthesis of new effective drugs prior to the synthesis of drugs. Quantitative structure-activity relationships (QSARs) are mathematical relationships linking chemical structure and pharmacological activity in a quantitative manner for a series of compounds. It is considered a major method of chemical research all over the world nowadays, and is frequently used in agricultural, biological, environmental, medicinal, and physical organic studies. Several investigations have been carried out in order to improve on this subject (4–10). Mathematical models have been used to correlate chemical structure with biological activities/properties. QSAR has great potential for modelling and designing novel compounds with robust properties by being able to forecast physicochemical properties as a function of structural features. 

The main purpose of QSAR studies is to instate an empirical rule or function relating to the descriptors of compounds under the investigation of activities or properties. This rule of function is then utilized to predict the same activities of the compounds not involved in the training set from their descriptors. The activity that can be predicted with satisfactory accuracy depends on a great extent on the performance of the applied multivariate data analysis method, which has provided the property being predicted, and is related to the descriptors (11). Model development in QSAR studies comprises different critical steps, such as: 1) Molecular Structures, 2) Molecular Descriptors, 3) Data Pre-processing, 4) Multivariate Analysis, and 5) Statistical Evaluation. Among the investigation of QSAR, one of the most important factors affecting the quality of the model is a method to build the model.

The traditional approach to QSAR relies heavily on multiple linear regressions (MLR). MLR analysis fails to give accurate results in the presence of collinear variables, or when the number of descriptors is large compared with the number of molecules, whereas by orthogonalization of the variables into low dimensional space, the factor analysis-based methods—such as principal component regression (PCR) and partial least square (PLS)—can overcome the drawbacks encountered for MLR (12). 

The theory of PCR and PLS, and its application in QSAR, are reported by several of the workers (13–18). Since it is not possible to know a priori which molecular properties are most relevant to the problem at hand. PLS, like other modelling methods, is often used in conjunction with optimization techniques for feature selection (19). One of the best methods for variable selection is genetic algorithms (GAs) (20–24). Genetic algorithms is a stochastic method for optimization based on the evolution process of living beings in which simplicity and effectiveness have been applied to the various types of optimization problems in many scientific fields (25). Accordingly, the combination of Quantitative Structure Activity Relationship (QSAR) and multivariate image analysis techniques, which are briefly called MIA-QSAR, is used to estimate the activity of various drugs. Sarkhosh *et al.* used genetic algorithms for the variable selection in the MIA-QSAR (11).

The present paper is focused on the application of 2D images, which are the proper structures of the compounds that can be drawn with aid of any appropriate program, as descriptors in QSAR. These images (2D chemical structures) have shown excellent correlation with bioactivities, and are supposed to codify chemical properties like size of substituents, chains, branches, and chiral centres. However, as far as we are aware, the MIAQSAR/QSPR process involves manual structural drawing and alignment, which can result in imperfections, *i.e.* common structural parts along with the congeneric series may not be exactly congruent and, therefore, spurious variances are inserted in the calibration step, reducing the model’s reliability and accuracy. Therefore, feature selection can minimize such effects by eliminating undesirable descriptors as well as those collinear ones (26).

## Experimental

*Data set*

The chemical structures of the HEPT are shown in Table 1. The RT inhibition data are reported according to Reference 27.

*Multivariate image analysis*

**Figure 1:**
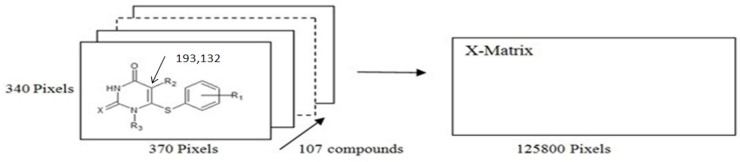
2D images and unfolding step of the 107 chemical structures to give the X-matrix. The arrow in structure indicates the coordinate of a pixel in common among the whole series of compounds, used in the 2D alignment step

MIA structures are 2D images that can be drawn with the help of some chemical Structure-drawing software. Accordingly, the 107 molecules that constitute the dataset were modelled using the ChemSketch program, and each file was saved as bitmaps in the Paint application of Microsoft Windows in a workspace of 370 × 340 pixel size (example of how chemical structures were drawn is given in Figure 1). In our dataset, the pixel located at the 193 × 132 coordinate (common to the whole series) was used as reference in the alignment step. Each 2D image was read and converted into binaries (double array in MATLAB), where black pixels are 0 and white pixels (where there is no chemical structure drawn) are 765, according to RGB colour composition. Each image of dimension 370 × 340 pixels was unfolded to a 1 × 125,800 row and then the 107 images were grouped to form a 107 × 125,800 matrix. Many columns do not have variance, because they correspond either to blank workspace or congruent structures and, therefore, they can be removed. This process gave a matrix of 107 × 9866 size and all completely similar descriptors for all molecules are deleted and finally the number of descriptors is reduced to 1254 then all pixel data are mean-centred. To build and validate the QSAR model, the study dataset is divided into a training set and a test set. The probability of overfitting of the model increases by selection of a series of similar molecules in the training set. To ensure that training and test sets cover the whole area of the dataset, it is divided into the parts of training and prediction sets, according to the Kennard-Stones algorithm. Kennard-Stones programs were written in MATLAB in accordance with the algorithm (28). The training set includes 91 compounds and the prediction set includes 16 compounds. The Kennard-Stones algorithm is known as one of the best ways of building training and prediction sets, and has been used in many QSAR studies.

Also, for the evaluation of the predictive ability of a different model, the root mean square error of prediction (RMSEP) and relative standard error of prediction (RSEP) can be used:

$\mathrm{RMSEP}=\sqrt[{2}]{\frac{\sum_{i=1}^{n}{\left(y_{i,\mathrm{pred}}-y_{i,\mathrm{obs}}\right)}^{2}}{n}}$

$\mathrm{RSEP}(\%)=100\times \sqrt[{2}]{\frac{\sum_{i=1}^{n}{\left(y_{i,\mathrm{pred}}-y_{i,\mathrm{obs}}\right)}^{2}}{n\sum_{i=1}^{n}{\left(y_{i,\mathrm{obs}}\right)}^{2}}}$

Where y_i,pred_ is the predicted activity using a different model, y_i,obs_ is the observed value of the activity, and *n* is the number of compounds in the prediction set.

*Genetic algorithm*

A genetic algorithm is a stochastic optimization method that has been inspired by evolutionary principles. GAs has five basic steps: 

1) An initial population of chromosomes is created. Each individual of the population, defined by a chromosome of binary values, represents a subset of the descriptors. The number of the genes in each chromosome was equal to the number of the descriptors. The population of the first generation was selected randomly. A gene was given the value of one, if its corresponding descriptor was included in the subset; otherwise, it was given the value of zero. The number of the genes with the value of one was kept relatively low to have a small subset of descriptors (29). 

2) The fitness of each chromosome in the population is evaluated by the predictivity of the model derived from the binary bit string. The n selected descriptors in each chromosome were evaluated by fitness function of the PLS, based on the following equation:

$\mathrm{Fitness}=\sqrt[{2}]{\frac{\mathrm{CUMPRESS}}{m-n}}$

where CUMPRESS and m are the cumulative predictive sum of square error and the number of compounds in the dataset respectively (30).

3) The population of chromosomes in the next generation is reproduced. The chromosome with the highest fitness is chosen as the best chromosome. 

4) The next step is a crossover such that each parent contributes a random selection of half of its descriptors and the offspring is constructed by combining these two halves of genetic code. 

5) Create next generation by combining and mutating the reproductive population and the new population. The best chromosome in the reproductive population is kept from the mutation process. Loop the steps 3–5 until a required termination criterion is satisfied.

The final model obtained is further refined by removing descriptors that do not significantly affect predictive accuracy. The cross-validation technique was used for evaluating the descriptors selected by GAs in each step.

Default values of the GAs program-as written by Leardi-were applied to most of the adjustable parameters of GAs, as listed in Table 2. The MATLAB 7.13 software was used to run the GA-PLS method, developed by Leardi (31). All descriptors by mean-centring before performing the GA-PLS were performed.

## Results and Discussion

*Principal component analysis of the data set*

Principal Component Analysis is a variable reduction procedure. Principal Components (PCs) are able to detect internal relations between characteristics of a set of objects, thus enabling a drastic reduction of the dimensionality of the original raw data. This reduction is achieved by transforming the original matrix to a new one, whose set of variables-termed PCs-appear to be orthogonal to each other (uncorrelated) and ordered so that the first few, with descending importance, retains most of the variance content from the total set of original variables (32).

**Table 1:** Chemical structures with the observed values of the anti-HIV activity for the HEPT derivatives (log (1/C50)).

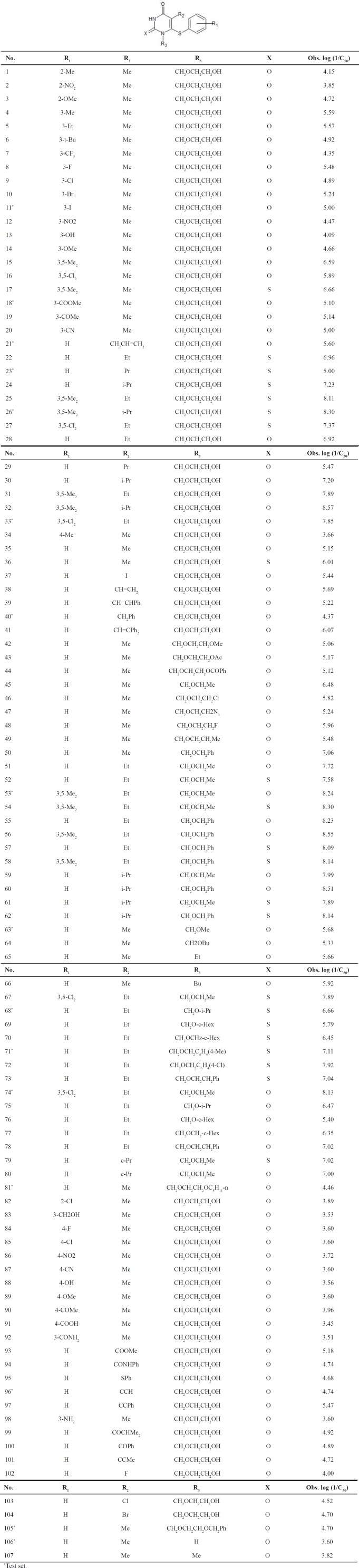

**Table 2:** Parameters of the genetic algorithms

**Parameter** *	**Value**
Population size	30 chromosomes
Response	cross-validated% explained variance
Maximum number of variables selected in the same chromosome	30
Probability of mutation	1%
Number of runs	100
Window size for smoothing	3

*On average, five variables per chromosome in the original population and backward elimination after every 100th evaluation and at the end.

**Table 3:** Observation and calculation values of activity using PCR, PLS and GA-PLS models

**Number of compounds (** Table 1 **)**	**Observation activity**	**PCR**		**PLS**		**GA-PLS**	
		**Predicted**	**Error (%)**	**Predicted**	**Error (%)**	**Predicted**	**Error (%)**
11	5.00	5.23	4.60	5.19	3.80	5.06	1.20
18	5.10	5.36	5.09	5.29	3.72	5.12	0.39
21	5.60	5.03	-10.17	5.11	-8.75	5.54	-1.07
23	5.00	4.23	-15.40	4.39	-12.20	4.96	-0.80
26	8.30	8.68	4.57	8.51	2.53	8.33	0.36
33	7.85	8.06	2.67	7.94	1.14	7.81	0.51
40	4.37	4.01	-8.23	4.16	-4.80	4.32	-1.14
53	8.24	8.75	6.19	8.34	1.21	8.26	0.24
63	5.68	5.93	4.40	5.88	3.52	5.72	0.70
68	6.66	6.86	3.00	6.84	2.70	6.81	2.25
71	7.11	6.56	-7.73	6.72	-5.48	7.14	0.42
74	8.13	8.69	6.88	8.57	5.41	8.09	-0.49

**Table 4:** Comparison of the statistical parameters by different QSAR models for the prediction of the activity

**Methods**	**Data set**	**R** **2**	**Q** 2*
PCR	Training	0.7929	0.7812
	Test	0.7822	0.7346
PLS	Training	0.8427	0.8109
	Test	0.8126	0.8033
GA-PLS	Training	0.9412	0.9371
	Test	0.9208	0.9124

*Q2 coefficient for the model validation by leave-one-out.

**Table 5:** Comparison between some works on the same set of HEPT derivatives

**Model**	**R** **2**	**Q** **2**	**NF** *	**Reference**
MLR	0.900	0.745	9	
				(34)
PLS	0.889	0.860	9	
MLR	0.815	0.783	5	
				(35)
MLR	0.811	0.778	6	
NN	0.919	0.779	6	
				(36)
MLR	0.856	0.814	4	
NN	0.850	0.878	4	
				(37)
SVM	0.874	0.867	4	
PCR	0.793	0.781	7	
PLS	0.842	0.812	5	This work
GA-PLS	0.941	0.937	3	

*Number of factor (Latent variables).

**Table 6:** Chemical structures with the observed values of the anti-HIV activity for the HEPT derivatives

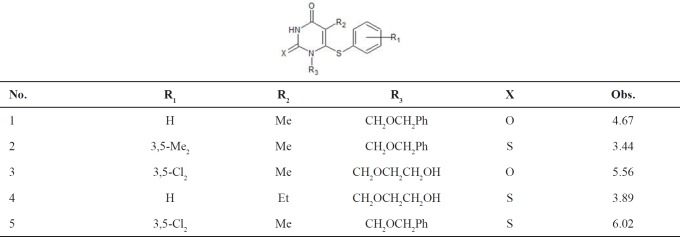

**Figure 2:**
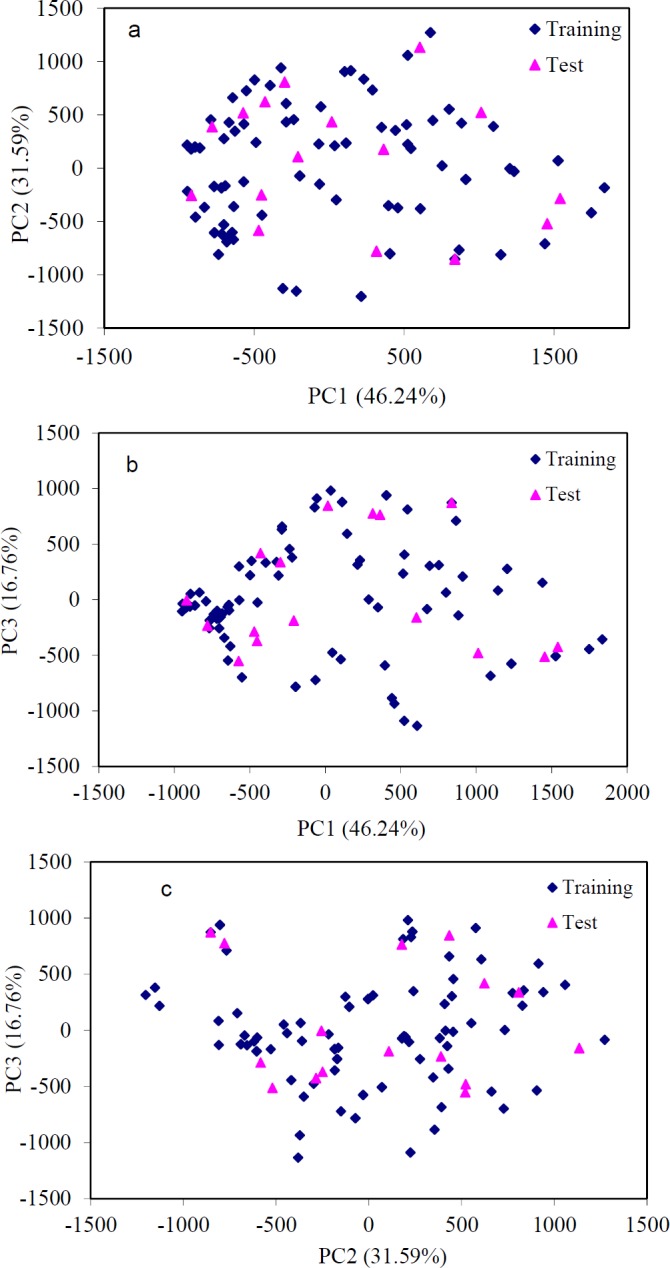
Principal components analysis of the 2D image descriptors for the data set, (a) PC2 versus PC1, (b) PC3 versus PC1 and (c) PC3 versus PC2

**Figure 3:**
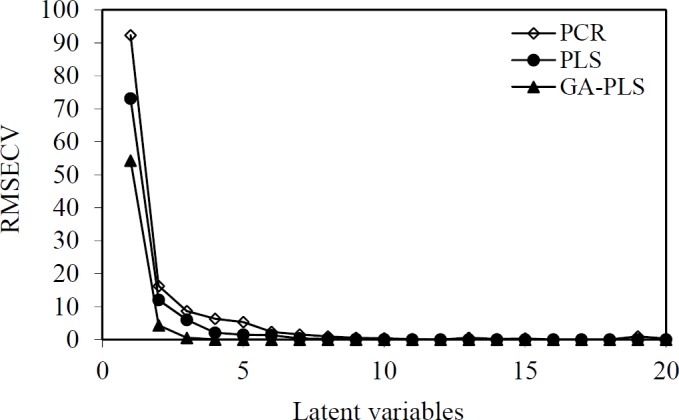
The RMSECV versus number of latent variables

**Figure 4:**
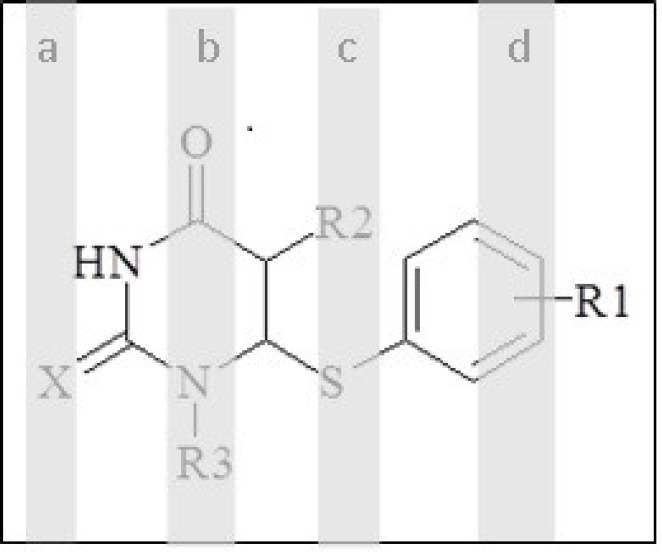
Selected regions by genetic algorithms

Accordingly, PC1 is defined in the direction of maximum variation of the whole dataset. PC2 is the direction that describes the maximum variance in the orthogonal subspace to PC1. The PCA was performed with the calculated structure descriptors for the whole dataset to detect the homogeneities in the dataset, and also to show the spatial location of the samples to assist the separation of the data in the training and test sets. The PCA results showed that three principal components (PC1, PC2, and PC3) described 94.53% of the overall variables, as follows: PC1 = 46.24%, PC2 = 31.59% and PC3 = 16.76%. Most of the variance is accounted for in the 3 first PCs. Their score plot is a reliable presentation of the spatial distribution of the points in the dataset. As can be seen in Figure 2, there is no clear clustering between compounds. The data separation is very important in the development of reliable and robust QSAR models. The quality of the prediction depends on the dataset used to develop the model. For regression analysis, the dataset was separated into two groups, a training set (91 data) and a prediction set (16 data), according to the Kennard-Stones algorithm. As shown in Figure 2, the distribution of the compounds in each subset seems to be relatively well-balanced over the space of the principal components.

*PCR and PLS modeling*

The general purpose of the linear regression method is to quantify the relationship between several independent or predictor variables and a dependent variable. PLS is a linear modelling technique where the information in the descriptor matrix X is projected onto a small number of underlying (‘latent’) variables called PLS components or latent variables. The matrix Y is simultaneously used for the estimation of the ‘latent’ variables in X, which will be the most relevant for the Y variables prediction. Independent or predictor variables could cause pixel changes in descriptors of image of molecules, their principal components or latent variables. In multivariate calibration, such as PCR and PLS models, a predictive model can be obtained by selecting the optimum number of components using a cross-validation technique. In the cross-validation technique, one or more samples in the dataset are omitted, and the rederived PLS model is used to predict the biological activity of the omitted samples. This process is repeated until the biological activity of all samples in the dataset has been predicted once. The number of principal factors (nLV) of PLS is an important parameter in the modelling. The parameter is determined on the basis of assessing root mean square error of calibration (RMSEC) and root mean square error of cross validation (RMSECV). The number of PLS factors included in the model was chosen in accordance with the lowest RMSECV. As shown in Figure 3, the RMSECV is minimized when the value of LVs is 7 and 5, and thus, the optimum LVs for the training set of PCR and PLS methods were respectively chosen to be 7 and 5. Prior to the PCR and PLS analysis, the dataset was mean-centred.

*GA-PLS modeling*

In the multivariate imaging analysis, the number of dependent variables is very large, so data reduction is very necessary. To find the more convenient set of descriptors in PLS modelling, genetic algorithms were used. The hybrid method that integrates GA as a powerful optimization tool and PLS as a robust statistical tool are applied to variable selection and modelling. After the running of GAs for pixel variables, the selected pixel descriptors were used for the running of PLS. When GA-PLS was used, the number of latent variables reduced to 3 (Figure 3). In each feature selection method, the variables remaining after the exclusion of non-significant parameters were cross-correlated in order to select the most relevant parameters concerning the following criteria: 1) *p <*0.05; 2) having the highest correlation with experimental data; and 3) having the lowest correlation with each other (33). The range of selected pixel descriptors is shown in Figure 4. According to the descriptors selected by genetic algorithms, it was found that the maximum structural effects are in a, b, c, and d regions (Figure 4). It seems that regions b, c, d—due to having the different functional groups—have a greater impact on the anti-HIV activity. This is because substituting O or S instead of X in the region a does not have a large impact on response. Selected areas in all the molecules are not identical in structure.

*Model validation and prediction of anti-HIV activity*

In Table 3, the predicted values of activity obtained by the PCR, PLS and GA-PLS methods and the per cent relative errors of prediction are presented. The data observed and predicted activity for GA-PLS are distributed about a straight line with the corresponding slope and intercept equal to 0.9987 and 0.0085 respectively, which are nearly close to the perfect values: one and zero, correspondingly. The relative errors of prediction are between -1.14% and 2.25%. This was obtained by using the GA-PLS method, which shows the high-quality predictive capability of the developing QSAR model. The data presented in Table 3 indicate that the GA-PLS model has good statistical quality with low prediction errors, while the GA-PLS model uses fewer latent variables. 

Table 3 also shows RMSEP and RSEP to predict the activity of anti-HIV activity. Other statistical parameters have been to evaluate the suitability of the models developed for predicting the activity of the studied compounds, and this includes cross validation coefficient (Q^2^ and R^2^). An inspection of the results of the table reveals higher R^2^ and Q^2^ values and lower RMSCEV and RMSEP for the GA-PLS method compared with their counterparts. These results showed GA-PLS is significantly better than that of the other models. These parameters are listed in Table 4, and show good statistical qualities. 

The results were summarized and compared to the other models obtained by some works on the same set of HEPT derivatives in Table 5. These results suggest the MIA-QSAR method is a useful tool, as promising as the most refined widely applied 2D methodologies, to correlate real pIC50 with pIC_50_ provided by descriptors from modelled structures for this series of anti-HIV compounds. Also, this comparative table makes it clear that MIA is at least as predictive as these 2D refined methodologies, being, therefore, a much less expensive alternative to propose new HETP derivatives, since MIA-QSAR showed a Q^2^ superior to all models available in the literature for this series of compounds.

*Molecular design*

As an application of the proposed method, we investigated GA-PLS model to predict the anti-HIV activity of five new HETP compounds on which biological tests were not performed yet. 

Table 6 shows the chemical structure of five new HETP compounds and their activity calculated by this proposed method. According to GA-PLS model, we have found the new HEPT 5 molecules (Table 6).

## Conclusion

In the present study, the multivariate image analysis descriptors used in quantitative structure-activity relationships are direct representations of chemical structures as they are simply numerical decodifications of pixels forming the 2D chemical images. This method allows the application of free drawing software and well known multivariate regression algorithms, such as PLS. In addition, it does not require conformational screening and 3D alignment, but only a 2D alignment step, which is simpler and faster than the current three-dimensional procedures. The combination of PLS analyses and genetic algorithms (GA-PLS) is used to develop a regression technique, the hybrid approach that integrates GA as a powerful optimization tool and PLS as a robust statistical method. These are applied to variable selection and modelling. A comparison of the results obtained by GA-PLS and the other regression methods utilized indicates higher accuracy of this method in describing anti-HIV activity of the HETP derivatives. The MIA descriptors can be used to make useful predictions, which is exceedingly useful for those who are designing and synthesizing more new active species. Moreover, the MIA-QSAR technique provides chemical information since, depending on the way in which substituent groups are drawn, they can encode steric effects. The QSAR model developed in this study can provide a useful tool to predict the activity of new compounds and also to design new compounds with high activity.
